# Translation and psychometric validation of the Persian Care Evaluation Scale-2 short version in bereaved family members of cancer patients

**DOI:** 10.1016/j.apjon.2025.100790

**Published:** 2025-09-23

**Authors:** Hosein Mohammadi Roshan, Mahmood Salesi, Mobina Golmohammadi, Salman Barasteh

**Affiliations:** a Student Research Committee, Baqiyatallah University of Medical Sciences, Tehran, Iran; b Chemical Injuries Research Center, Systems Biology and Poisonings Institute, Baqiyatallah University of Medical Sciences, Tehran, Iran; c Nursing Care Research Center, Clinical Sciences Institute, Baqiyatallah University of Medical Sciences, Tehran, Iran

**Keywords:** Bereavement, Cancer, Family caregivers, Hospice, Palliative, Quality of care

## Abstract

**Objective:**

To translate and evaluate the psychometric validation of the Persian version of the Care Evaluation Scale-2.0 Short (CES2.0-S) for bereaved family members of cancer patients.

**Methods:**

This methodological study was conducted in Tehran in 2024. The scale was translated using forward–backward translation. Face validity was assessed through cognitive interviews with 10 bereaved family members, and content validity was evaluated by 10 palliative care experts using the Content Validity Index. Construct validity was examined through exploratory factor analysis and convergent validity. Reliability was assessed by internal consistency and test–retest stability over a two-week interval.

**Results:**

A total of 237 bereaved family members participated. Exploratory factor analysis identified two factors— support provided by the care team and access to healthcare services —explaining 63.4% of the total variance. The Persian CES2.0-S demonstrated strong internal consistency (Cronbach's α ​= ​0.89) and test–retest reliability (ICC ​= ​0.80). Convergent validity was supported by significant correlations with the Good Death Questionnaire-Short Form (*r* ​= ​0.47, *P* ​< ​0.001) and satisfaction with end-of-life care (*r* ​= ​0.48, *P* ​< ​0.001).

**Conclusions:**

The Persian CES2.0-S is a valid and reliable instrument for assessing structures and processes of end-of-life care among bereaved family members of cancer patients. Its use may help identify areas requiring improvement and guide strategies to enhance the quality of end-of-life care, an essential goal of palliative care.

## Introduction

Palliative care requires creative approaches to reduce suffering and increase quality of life, enabling patients to address their physical, psychosocial, and spiritual concerns while maintaining human dignity.^1^ One of the responsibilities of caregivers specializing in palliative care is to strive to improve both the quality of life and the quality of end-of-life care (EoLC).^2^

Cancer is the second leading cause of death in the worldwide^3^ and the third leading cause in Iran.^4^ In 2020, approximately 10 million people died from cancer, accounting for almost one in six deaths worldwide.^5^ According to the World Health Organization (WHO), 13 million people are expected to die from cancer by 2030.^6^ In Iran an estimated 90,000 new cases of cancer are registered annually.^4^ The country is currently undergoing an epidemiological shift from communicable to non-communicable diseases, including cancer, and is facing a doubling of its disease burden.^7^

Studies have shown that perceptions of end-of-life care can vary among patients, caregivers, physicians, and relatives.^8^, ^9^, ^10^, ^11^ In the final stages of life, patients are often unable to provide feedback on the perceived quality of care. However, their loved ones can evaluate the care provided during the patients’ final days.^12^ Therefore, the availability of a reliable tool to assess the EoLC from the perspective of loved ones is essential for delivering comprehensive and patient-centered EoLC.^13^

To date, numerous tools have been developed to assess the quality of end-of-life care. Some of the most widely used and significant instruments include: Quality of Dying and Death (QODD),^14^ Quality Of Dying In Long-Term Care (QOD-LTC),^15^ Good Death Inventory (GDI),^16^ Views Of Informal Carers – Evaluation Of Services (Short Form) (VOICE-SF),^17^ Evaluating Care And Health Outcomes-For The Dying (ECHO-D),^18^ Quality Care Questionnaire–End Of Life (QCQ-EOL)^19^ Toolkit After-Death Bereaved Family Member (TIME),^20^ Quality Of End-Of-Life Care And Satisfaction With Treatment (QUEST),^21^ Care Evaluation Scale (CES1.0),^22^ Problems And Needs In Palliative Care Questionnaire (PNPC),^23^ Mcgill Quality Of Life Questionnaire (MQOL),^24^ and Care of the Dying Evaluation- German Version (CODE-GER).^25^

The CES is one of the key tools developed to assess the quality of EoLC, particularly in terms of evaluating the structure and process of care from the family's perspective. While other tools focus primarily on patient outcomes or symptom burden,^26^^,^^27^ but CES comprehensively addresses dimensions of care delivery, including coordination, access, and support for family decision-making.^22^ This scale was specifically designed to reflect the core dimensions of quality palliative care, based on empirical research and expert opinion.^22^^,^^28^ The initial version of the CES, developed by Morita et al., in 2004, provides a robust practical and psychometric framework for identifying areas of improvement in EoLC.^22^ In 2017, Miyashita et al. revised the original CES and developed the CES2.0, as well as its short form (CES2.0-S). The revision aimed to reduce misresponses while maintaining strong reliability and validity.^28^ The short version (CES2.0-S) was introduced to further adaptable to clinical and research settings, making it particularly suitable for large-scale assessments or low-resource settings.^28^ These features make CES a well-known and widely used tool in palliative care research and practice.

Palliative and EoLC in Iran is still in its infancy and is available only in a few centers in large cities.^29^ Most people and their families prefer to receive care at home, but as death approaches, they tend to seek care in hospitals.^30^ The expansion of palliative care in Iran faces challenges such as weak governance, poor infrastructure, limited public awareness, and inadequate drug supply,^29^ highlight the necessity of a tool that examines the process and structure of providing the EoLC. In such circumstances, researchers have two options: designing a new questionnaire that has a long process and requires adherence to specific scientific and specialized principles, or using existing (foreign) questionnaires whose validity and reliability have already been confirmed.^31^ Therefore, this study was conducted to translate and psychometric validation of the Persian CES2.0-S in Iran.

## Methods

### Study design

This methodological study was conducted in 2024 with the aim of translating and examining the psychometric validation of the Persian CES2.0-S in bereaved family members of cancer patients at Baqiyatallah Hospital in Tehran.

### Study population/ sampling

**Fig. 1:**
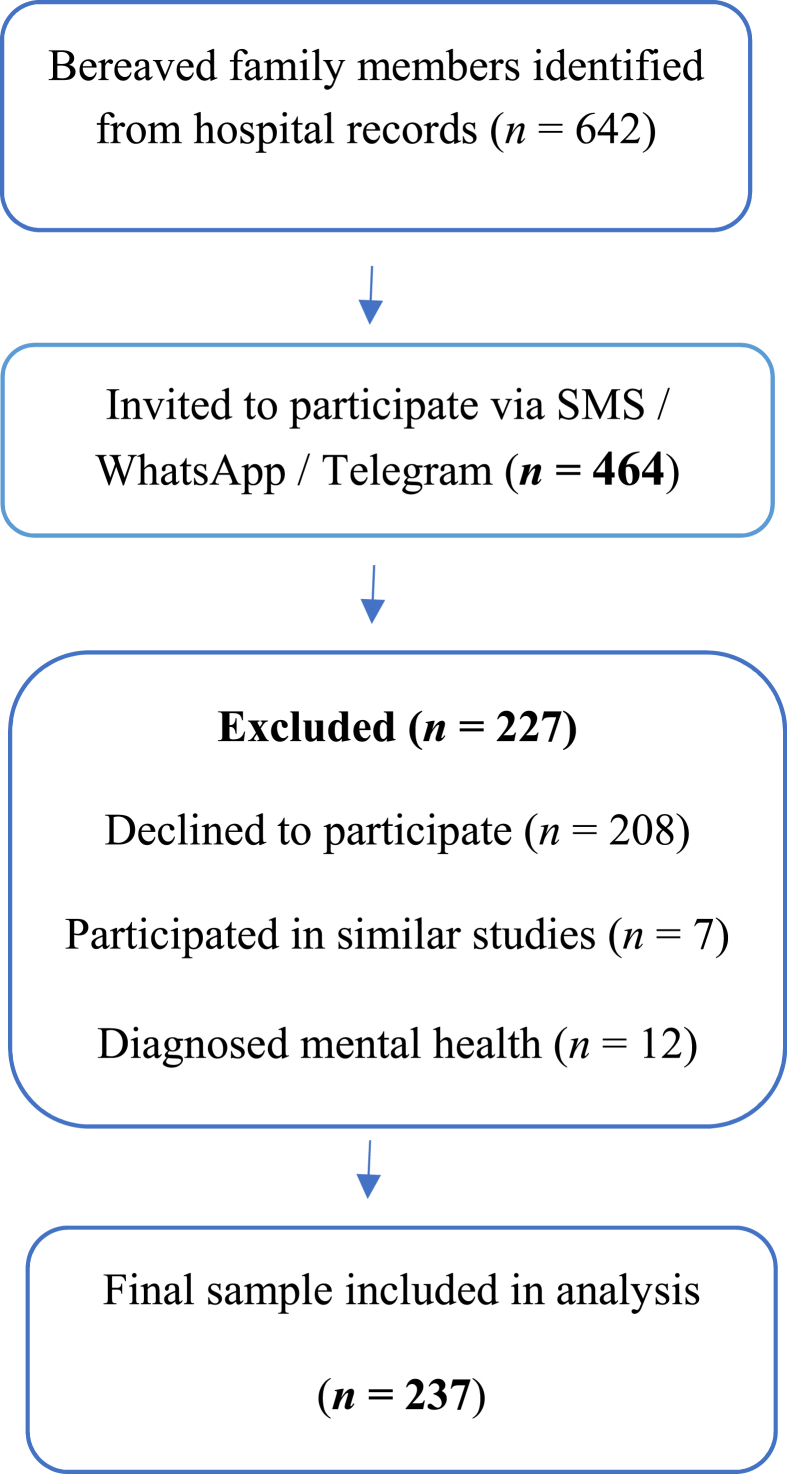
Participant flow chart.

In this study, family caregivers of cancer patients who died at Baqiyatallah Hospital were selected as the research population by convinence sampling. This approach was chosen due to the difficulty of reaching bereaved families, largely due to their challenging emotional circumstances.^32^ For each deceased patient, only one family member was invited. Inclusion criteria included a definitive diagnosis of cancer of the deceased, at least twenty years of age of the deceased, willingness to participate in the study by the family member, ability to respond to a self-report scale, family member's awareness of the diagnosis of malignancy, and the ability to communicate with bereaved family members via SMS or WhatsApp and Telegram platforms. Social media platforms are widely used and easily accessible among the general Iranian population, including family caregivers, and were therefore utilized for collect data. These platforms facilitated efficient, cost effective, and timely communication with geographically dispersed individuals.^33^ Exclusion criteria included unwillingness to continue cooperation of family caregivers in completing the scale or incomplete completion of the scale, participation of participants in other studies with the same theme, and mental disorders of the participants. In this study, 464 bereaved family members were contacted, of which 237 completed the questionnaire (response rate 51.1%) (Fig. 1).

### Study instrument

#### Demographic information sheet

Demographic information collected from participants included age, gender, education level, relationship to the patient, and income. Family members were also asked about their overall satisfaction with end-of-life care.

#### Care Evaluation Scale 2- short version

The 10-item Care Evaluation Scale 2.0 developed based on the original 28-item CES2.0, which itself was a revised version of the initial CES1.0, designed to evaluate the structure and process of end-of-life care from the perspective of bereaved family members. The full CES2.0, was developed for bereaved family members based on modifications of the original CES1.0, whose validity and reliability were examined in a study by Miyashita et al., in 2017.^28^ In this scale, participants are asked to answer 28 items using a seven-point Likert scale (7: not applicable, 6: strongly agree, 5: agree, 4: somewhat agree, 3: somewhat disagree, 2: disagree, 1: strongly disagree) to assess the structure and process of end-of-life care. For ease of interpretation, all scores are proportionally adjusted in the range of 0–100. Higher scores indicate a good care structure or process. To establish construct validity, an exploratory factor analysis was conducted, identifying 10 subcategories: physical care by the physician, physical care by the nurse, psycho-existential care, explanation to the patient by the physician, explanation to the family by the physician, environment, cost, attention to family health, accessibility, coordination, and adaptation. Reliability was assessed using a Cronbach's alpha coefficient of 0.96 and an intraclass correlation coefficient (ICC) of 0.83. In addition, the CES2.0 showed a strong correlation with overall satisfaction (*r* ​= ​0.83) and a moderate correlation with the Family Satisfaction with Advanced Cancer Care (FAMCARE) questionnaire (*r* ​= ​0.58). It also showed negative correlations with the Patient Health Questionnaire-9 (PHQ-9; *r ​=* ​−0.22) and the Brief Bereavement Questionnaire (BGQ; *r ​=* ​−0.10). To improve usability in research and clinical settings, a short version comprising 10 key items was developed. In the current study, this brief version was administered to bereaved family members using the same seven-point Likert scale format.^28^

#### Good death inventory- short form

The Good Death Inventory (GDI) was designed by Miyashita et al., in 2008^16^ to investigate factors associated with a good death in people with cancer. The short form of this instrument consists of 18 items covering ten main areas and eight sub-domains. These divided factors reflect good death from the perspective of patients' family members and include ten main areas: physical comfort, dying in a favorite place, maintaining hope and pleasure, having good relationship with medical staff, not being burden, having good relationship with family, independence, environmental comfort, being respected as a person, and a sense of life completion. The eight sub-domains are: receiving adequate treatment, natural death, preparation for death, future control, ignorance of death, pride and beauty, feeling that one's life is worth living, religious and spiritual comfort. These items are completed by family members of patients with cancer. The reliability of the instrument was calculated using the test-retest method, yielding an ICC of 0.52 and also Cronbach's alpha ranged from 0.74 to 0.95.^16^ The translation and psychometric testing of the short form of this questionnaire were conducted in Persian by Mohammadi Roshan et al., in 2023^34^ using a sample of families of deceased cancer patients. 4 items were removed in an exploratory factor analysis in the Iranian population, resulting in a final version consisting 14 items. The response is recorded on a seven-point Likert scale (1 ​= ​strongly disagree, 2 ​= ​disagree, 3 ​= ​somewhat disagree, 4 ​= ​not sure, 5 ​= ​somewhat agree, 6 ​= ​agree, 7 ​= ​strongly agree). The items are classified into three categories: peace, hope, and the value and quality of treatment. Cronbach's alpha coefficient of the questionnaire is 0.84, and the stability reliability was confirmed with an ICC of 0.85.^34^ The Good Death Inventory-Short Form (GDI-SF) was chosen because its Persian version has been psychometrically validated. The Persian version has 14 items and primarily focuses on the quality of end-of-life care from the perspective of bereaved families. Additionally, the limited number of items facilitates participants' responses. These features make the GDI-SF an appropriate option for assessing the convergent validity of the Persian CES2.0-S.

### Translation procedure

The translation process was conducted using Brislin's dual translation model.^35^ Initially, the researchers obtained permission from the developer, Miyashita,^28^ to translate and evaluate the psychometric validation of the scale into Persian. During the forward translation stage, two bilingual translators—one expert fluent in English and a nurse familiar with palliative care and end-of-life concepts—independently translated the tool into Persian. The translated versions were then synthesized into a single questionnaire, which was reviewed, edited, and approved in a meeting with experts and an English language specialist. In the backward translation stage, two native English speakers fluent in Persian, who were blinded to the original scale, independently translated the Persian version back into English to ensure accuracy and preservation of the original meaning. The final English version was sent to the scale developer for confirmation. After receiving approval, the finalized Persian version was prepared for use in the study (Appendix A).

### Face validity

After the translation process was completed, face validity was assessed by cognitive interviews with the participants. Cognitive interviewing is used to identify potential sources of error in the questionnaire by focusing on the cognitive process's respondents engage in while completing it.^36^ The qualitative face validity of the scale was assessed by face-to-face interviews with 10 family members of the patients, who participated only for the purpose of evaluating validity. The scale was provided to them, and they were asked to share their opinions regarding the difficulty, understanding, appropriateness and relevance of the items to the dimensions of the scale, as well as the potential for misinterpretation or inadequacy in the meanings of the words. In the end, no changes were made to the items due to their simplicity.

### Content validity

Content validity is assessed to ensure that all important aspects of the intended concept of the scale, as well as to determine the acceptability and generalizability of its implementation by experts.^37^ In this study, content validity was evaluated through interviews with experts and calculation of the Content Validity Index (CVI). The Persian CES2.0-S was provided to 10 experts in the field of palliative care, to assess the relevance each item based on a four-point Likert scale (1 ​= ​not at all relevant; 2 ​= ​somewhat relevant; 3 ​= ​fairly relevant; 4 ​= ​completely relevant). The CVI score was then calculated for each item. A CVI score above 0.79 is considered appropriate, a score between 0.70 and 0.79 indicates that the item requires correction and revision; and a score below 0.7 is unacceptable and should be eliminated.^38^ Additionally, ceiling and floor effects were calculated. The presence of ceiling and floor effects indicates insufficient content validity. Specifically, if more than 15% of participants obtain the highest or lowest possible score, a ceiling or floor effect is said to be present, respectively.^39^

### Item analysis

This stage represents the initial evaluation of the scale within the target population and is carried out prior to assessing construct validity. As part of this phase, inter-item correlations were examined. Items with a correlation coefficient of less than 0.32 or greater than 0.9 with at least one other item were removed. The analysis included Cronbach's Alpha, inter-item correlation, Cronbach's Alpha if item deleted, and item–total correlation.^40^

### Construct validity

To examine the construct validity of this scale, two methods were used: exploratory factor analysis (EFA) and convergent validity.

#### Exploratory factor analysis (EFA)

In the first step, the Keiser-Meyer-Olkin (KMO) test and Bartlett's test were performed to assess sampling adequacy and the suitability of the data for factor analysis. For factor analysis, a KMO value closer to 1 indicates better sampling adequacy; however, a value greater than 0.5 is consider acceptable, and a value above 0.7 is preferred.^30^ Bartlett's test is acceptable when the significance level is below 0.05.^41^^,^^42^ Favorable results from the both the KMO and Bartlett's tests indicate the existence of a desirable correlation matrix for conducting factor analysis.^43^ Factor loading represents the relationship between each factor and each item in the scale. In this study, a minimum factor loading of 0.3 was considered acceptable; value below 0.3 indicate a weak relationship between the item and the factor.^44^^,^^45^ In this study, considering the natural distribution of the data, the Maximum Likelihood (ML) method was used to extract factors, and varimax rotation was applied to ensure the interpretability of the factors.^46^

#### Convergent validation

Convergent validity, a subset of construct validity, examines whether the scores of the scale under study seem “reasonable” when compared with scores of other related scales. Scale scores are expected to correlate with those of other related measures to the extent theoretically anticipated.^47^ In this study, participants answered the Persian version of the Good Death Inventory-Short Form in addition to the main questionnaire. The correlation between the two instruments was examined using the Pearson correlation coefficient.^48^ Additionally, respondents answered a general question regarding their satisfaction with end-of-life care, and its correlation with the CES2-S was also assessed.

### Reliability

Reliability refers to the accuracy of the data obtained and the extent to which random error is controlled by the measurement tool.^49^ To determine the reliability and internal consistency of the CES2.0-S, a Cronbach's alpha coefficient of greater than 0.7 was considered acceptable internal consistency.^50^ Additionally, test–retest reliability was evaluated using a sample of 30 participants over a two-week interval. The ICC was calculated to assess stability with an ICC greater than 0.80 is considered a desirable level of stability.^51^

### Data analysis

SPSS version 26 software was used to analyze the data. Descriptive statistics included frequencies and percentages, while analytical statistics involved factor analysis with rotation, correlation analysis, Cronbach's alpha coefficient, and the ICC. The Kolmogorov–Smirnov test was used to assess the normality of the data, which was confirmed with a *P*-value of 0.2. Therefore, the Pearson correlation coefficient was used to evaluate convergent validity. A significance level of *P* ​< ​0.05 was considered statically significance for all analyses.

### Ethical consideration

Permission to conduct the study was obtained from the Ethics Committee of Baqiyatallah University of Medical Sciences (Approval No. IR.BMSU.BAQ.REC.1403.229). The data analysis process was carried out in accordance with the ethical standards of the 1964 Declaration of Helsinki and subsequent amendments or similar ethical standards.^52^ Written permission to use and translate the scale was obtained via email from the original tool developer prior to initiating the translation process. Informed consent was obtained from all participants. They were informed about the objectives of the study and were assured that their information would be kept confidential.

## Results

### Participant's characteristics

The Participants included 131 men and 106 women, with a mean age of 45.01 (± 11.59). The majority of participants (62.4%) were children of the deceased. Most had a university-level education (60.3%), and the majority of families reported a middle-income level (59.1%) (Table 1). To facilitate access to participants, the questionnaire was administered online. Following a phone call in which the purpose and procedures were explained, a link to the questionnaire was sent to the participants who provided verbal consent. The online questionnaire including demographic information, the Iranian version of the Good Death Inventory–Short Form, and Persian CES2.0-S. A total of 237 participants completed the online questionnaire.

**Table 1:** Demographic information of participants (*N* ​= ​237).

Variable	*n*	%
**Sex**		
Male	131	55.3
Female	106	44.7
**Age (years)**		
< 40	101	42.6
41-60	111	46.8
> 61	25	10.5
**Relation to the patients**		
Wife/husbands	52	21.9
Parents	3	1.3
Child	148	62.4
Other	34	14.3
**Education**		
Elementary	7	3.0
Middle school	18	7.6
Diploma	69	29.1
University education	143	60.3
**Income**		
High	2	0.8
Moderate	140	59.1
Low	95	40.1
**Satisfaction**		
High	41	17.3
Moderate	86	36.3
Low	110	46.4

### Procedure

The final of Persian CES2.0-S was obtained after Forward-Backward translation process, preparation of the English version, and approval by the original developer, Miyashita.

#### Face and content validity

Face validity was confirmed through cognitive interviews with 10 family members. To evaluate content validity, CVI was calculated for each item. All items scored above 0.79. The item-level CVIs (I-CVI) ranging from 0.8 to 1. The average CVI (S-CVI) across all items was 0.87, indicating acceptable content validity; therefore, no item was excluded at this stage. To examining the ceiling and floor effects, the minimum and maximum scores we identified, and the frequency of participants obtaining these scores was divided by the total number of participants. In this study, the ceiling effect was 1.2% and the floor effect was 4%, indicating no significant concern regarding extreme response distributions.

#### Item analysis

In the item analysis, no item had a correlation coefficient less than 0.32 or greater than 0.9.

#### Construct validity

##### Exploratory factor analysis

**Fig. 2:**
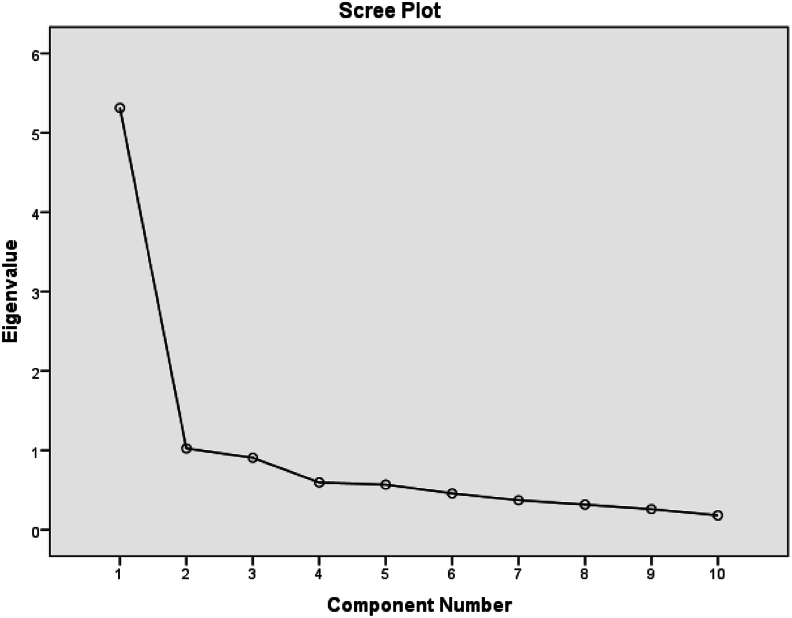
Scree plot.

A KMO value of 0.89 was obtained, indicating sampling adequacy, and Bartlett's test of sphericity was significant (χ^2^ ​= ​1264.66, df ​= ​45, *P* ​< ​0.000), confirming the suitability of data for factor analysis. Two factors were extracted and labeled (Table 2, Fig. 2). Two factors were: “Support Provided by the Care Team” (40.07% of the variance) and “Access to Healthcare Services” (23.32%). No items were removed from the scale at this stage.

**Table 2:** Exploratory factor analysis of the Persian CES 2-short version.

Factor	Items	Factor Loading%	Variance
Support provided by the care team	CES1	0.71	40.07%
	CES2	0.74	
	CES3	0.57	
	CES4	0.86	
	CES5	0.91	
	CES7	0.49	
	CES10	0.67	
Access to healthcare services	CES6	0.52	23.32%
	CES8	0.98	
	CES9	0.51	
**Cumulative**			63.39%

##### Convergent validation

For convergent validity, a significant and moderate correlation was found between the total scores of the Persian CES2.0-S and the Persian Good Death Inventory-Short Version (*r* ​= ​0.471, *P* ​< ​0.001). Additionally, statistical analyses showed a moderate correlation between the Persian CES2.0-S and overall satisfaction with end-of-life care (*r* ​= ​0.485, *P* ​< ​0.001).

#### Reliability

The reliability of the scale was assessed in terms of internal consistency and stability. Internal consistency, assessed using Cronbach's alpha coefficient, was 0.89 (0.66–0.94). Stability was performed using test-retest methods, and the ICC for the entire scale was 0.89. The ICCs for dimensions ranged from 0.79 to 0.89 (Table 3).

**Table 3:** Reliability.

Factor	Items	Alpha	ICC
Support provided by the care team	CES1, CES2, CES3, CES4, CES5, CES7, CES10	0.88	0.79 (0.23–0.82)
Access to health care services	CES6, CES8, CES9	0.67	0.89 (0.75–0.94)
Total		0.89	0.89 (0.66–0.94)

## Discussion

### Main findings

In the present study, the psychometric validation of the Persian CES2.0-S were evaluated among bereaved family members of cancer patients. The evaluation included face validity, content validity, and construct validity through EFA and convergent validity and reliability with internal consistency and test-retest methods. The results showed that the Persian CES2.0-S has appropriate validity and reliability for measuring the quality of EoLC from the perspective of family members of cancer patients. These findings are well consistent with the results of the original studies of the instrument developers.^22^^,^^28^

The translation process was carefully carried out until the final Persian version was achieved. One of the clear strengths of this study was the use of a standard translation method (Forward-Backward) and the review of the final scale by the original developer. This approach minimized the possibility of translation and transfer errors and ensured the accurate equivalence of concepts.^35^ Unlike the original study which did not conduct interviews, in this study the face validity of the scale using the opinions of 10 family members showed that the items were simple and clear and that none of the scale were subjected to any deletion of validities or factors during the translation and psychometric process. This indicates the clarity and conceptual fit of the items with the Persian language and culture and indicates that it is a scale that accurately covers the basis of quality EoLC.

Exploratory factor analysis identified two main factors: support from the care team and access to health services. These factors align with the theoretical structure of the scale and the findings of Miyashita et al.^28^

In the EFA, the two-factor model showed the best fit. The reduction in the number of items has changed the factor structure compared to the original multifactor version. These two new factors reflect key dimensions related to care evaluation in Iranian cultural and linguistic context. These two factors represent the most important dimensions of families' experience of end-of-life care, especially in situations where patients are no longer able to express their needs and expectations and families act as their representatives. The current 10-item scale with two factors explained a total of 63.39% of the total variance in the concept of evaluating EoLC to ensure comprehensive care for the patient. One of the main goals of factor analysis is to maximize explained variance; the cumulative variance reported in the study by Yusuke Kanno was 86.3%.^53^ Structural differences are likely due to translation, shortening of questions, sample characteristics, and cultural differences, which are natural and acceptable in the localization process of psychometric instruments.

In this study, reliability was confirmed by internal consistency with a Cronbach's alpha of 0.894 and stability assessed through test-retest and intracluster correlation test of 0.896, indicating appropriate reliability of the tool over time. In accordance with these findings in the study by Miyashita et al. (ICC ​= ​0.89) and Cronbach's alpha (0.83) were obtained.^28^ These results are also consistent with the findings of the study by Morita et al.^22^ which confirmed the validity and reliability of the Persian CES2.0-S. This issue is particularly important in Iran, where locally validated tools for measuring the quality of EoLC, especially from the perspective of families, are very limited. Therefore, the present study helps to fill this significant gap.

In this study, convergent validity was assessed to examine construct validity. In this method, participants also answered the Persian version of the good death inventory-short form, and the correlation between the two was measured using the Pearson correlation coefficient (*r* ​= ​0.485, *P* ​< ​0.001). Similarly, Yusuke Kanno used the short version of the CES to examine the concurrent validity of the Dying Care Process Scale for Bereaved Family Members, confirming its validity and reliability.^53^ Also, in this study, the total score of satisfaction with EoLC was positively correlated with the Persian CES2.0-S (*r* ​= ​0.485, *P* ​< ​0.001), which is similar to the original study.^28^ In a study by Tatsuya Morita et al., in 2004, the CES subscales were moderately correlated with the level of perceived experience and satisfaction (*r* ​= ​0.42 ​± ​0.69, *P* ​< ​0.01).^22^ These correlations support the good convergent validity of the scale and indicate that the scale not only has a sound conceptual structure but can also accurately reflect the quality of care in practice.

Given the increasing prevalence of cancer and the growing trend of aging in Iran,^4^^,^^6^ the need for palliative care and continuous assessment of the quality of these services is felt more than ever before. A valid and localized tool such as the Persian CES2.0-S can assist treatment teams, managers, and health policymakers in identify the strengths and weaknesses of their services and planning improvement in the quality of EoLC. Also, this scale can be used in future research as an indicator to evaluate the effectiveness of educational interventions, new policies, and quality improvement programs.

Finally, the results of this study showed that the Persian CES2.0-S is a valid and reliable tool for measuring the quality of EoLC from the perspective of families. Considering the small number of questions in this tool and the short time required to complete it, this tool can be an efficient tool in improving care processes and enhancing the quality of life of patients in the last days of life. It is recommended that future studies be conducted with larger and more diverse populations and in different centers to further examine the external validity of the tool and to allow for the generalization of the results.

### Implications for clinical practice and policy-making

This scale can be used in clinical settings to assess the standard of palliative care and identify areas where service delivery needs improvement. Due to its user-friendliness and small number of items, the scale is suitable for both high-volume hospitals and outpatient clinics. By using this tool, national initiatives to monitor and standardize palliative care across healthcare facilities can also be supported. Given the cultural context of Iran, where family participation in end-of-life decisions is highly valued, this method can provide evidence-based approaches to determine patient and family expectations of services. In addition, using the information collected from this scale, focused interventions and educational programs for doctors and nurses can be designed to improve the quality of hospital services.

### Limitations

One of the limitations of this study is that only examined the perspectives of the family members of patients at a single center (Baqiyatallah Hospital), which may limit the generalizability of the finding to the entire Iranian society. The reasons for choosing a single-center design at Baqiyatallah Hospital included the referral of patients from diverse socio-economic and cultural backgrounds in Tehran and surrounding areas, efficient coordination, consistent protocols and quality control of data collection for psychometric assessment, and better access to and communication with bereaved families through hospital-based support services. A diverse patient population attends Baqiyatallah Hospital. This diversity may reflect, to some extent, the broader Iranian population. However, future research focusing on hospitals in different geographical and cultural regions of Iran, along with additional external validation groups, is necessary to increase the robustness and generalizability of the CES2.0-S across the country. Sampling in this study was self-reported, and there is uncertainty about whether individuals tend to mention socially desirable opinions. Also, due to limited access to the families of bereaved patients and after death, the sampling method was carried out by convenience sampling. Another limitation was the use of social media platforms, which seem to be less used by individuals and the elderly due to their level of education and cognitive problems. Given that this study examined the perspectives of the families of patients who had died, one of the limitations was accessing the samples and obtaining their consent and convincing them to participate in the study, resulting in a response rate of 51%. The online sampling and 51% response rate may indicate that younger and more digitally literate individuals participated in the study, which suggests the possibility of selection bias.

## Conclusions

Based on the results, the Persian CES2.0-S demonstrates good validity and reliability. Therefore, this scale can assist the treatment team in managing symptoms, improving communication, and assessing the attitude of cancer patients toward receiving EoLC. Additionally, it enables families of cancer patients to provide a comprehensive assessment of the care provided at the EoLC. Furthermore, these scales may be useful in evaluating the effectiveness of care programs for cancer patients in their final stages, helping both the treatment team and families can improve the overall quality of EoLC.

## CRediT authorship contribution statement

**SB:** Conceptualization, Data curation, Investigation, Methodology, Writing - original draft, and Writing - review & editing. **HMR:** Methodology, Writing - original draft, and Writing - review & editing. **MS:** Methodology. **MG**: Conceptualization, Investigation, Writing - original draft, and Writing - review & editing. All authors have read and approved the final manuscript.

## Ethics statement

The study was approved by the Institutional Ethics Committee of Baqiyatallah University of Medical Sciences (Approval No. IR.BMSU.BAQ.REC.1403.229) and was conducted in accordance with the 1964 Helsinki Declaration and its later amendments or comparable ethical standards. All participants provided written informed consent.

## Data availability statement

The data are not publicly available due to restrictions their containing information that could compromise the privacy of research participants.

## Declaration of generative AI and AI-assisted technologies in the writing process

No AI tools/services were used during the preparation of this work.

## Funding

This research was supported by the 10.13039/501100005849Baqiyatallah University Medical Science. The funders had no role in considering the study design or in the collection, analysis, interpretation of data, writing of the report, or decision to submit the article for publication.

## Declaration of competing interest

The authors declare no conflict of interest.
