# Supplementary material for: Translation and psychometric validation of the Persian Care Evaluation Scale-2 short version in bereaved family members of cancer patients
Source: Asia Pac J Oncol Nurs. 2025 Sep 23;12:100790. doi: 10.1016/j.apjon.2025.100790 (PMC12538042; doi:10.1016/j.apjon.2025.100790)
Supplement: Multimedia component 1 [file mmc1.docx]

**Appendix A. Persian CES 2-Short Version**

| **غیر قابل اجرا** | **کاملا مخالفم** | **مخالفم** | **تا حدودی مخالفم** | **تا حدودی موافقم** | **موافقم** | **کاملا موافقم** | **گویه** |
| --- | --- | --- | --- | --- | --- | --- | --- |
|  |  |  |  |  |  |  | پزشک سعی داشت از درد جسمی بیمارم را بکاهد. |
|  |  |  |  |  |  |  | پرستار در تلاش بود تا درد جسمی بیمارم را تسکین دهد. |
|  |  |  |  |  |  |  | پزشکان، پرستاران و کارکنان سخت تلاش کردند تا از اضطراب و نگرانی بیمارم بکاهند. |
|  |  |  |  |  |  |  | توضیحات پزشک در مورد وضعیت بیمارم و جزئیات درمان کافی بود. |
|  |  |  |  |  |  |  | توضیحات پزشک در مورد وضعیت پزشکی و جزئیات درمان به خانواده کافی بود. |
|  |  |  |  |  |  |  | اتاق بیمارستان (خانه) برای زندگی مناسب و راحت بود. |
|  |  |  |  |  |  |  | مراقبت های لازم برای کمک به حفظ سلامت خانواده انجام شد. |
|  |  |  |  |  |  |  | مقدار هزینه های پرداخت شده معقول بود. |
|  |  |  |  |  |  |  | بیمارم می‌توانست در اسرع وقت در بیمارستان بستری شود. |
|  |  |  |  |  |  |  | همکاری بین پزشکان و پرستاران خوب بود. |

**CES 2-Short Version**

| **Item** | **Strongly agree** | **Agree** | **Somewhat agree** | **Somewhat disagree** | **Disagree** | **Strongly disagree** | **Not applicable** |
| --- | --- | --- | --- | --- | --- | --- | --- |
| The doctor tried to reduce my patient’s physical pain. |  |  |  |  |  |  |  |
| The nurse was trying to relieve my patient’s physical pain. |  |  |  |  |  |  |  |
| Doctors, nurses, and staff worked hard to reduce my patient’s anxiety and worry. |  |  |  |  |  |  |  |
| The doctor's explanations about my patient’s condition and treatment details were sufficient. |  |  |  |  |  |  |  |
| The doctor's explanations regarding the medical condition and treatment details were sufficient for the family. |  |  |  |  |  |  |  |
| The hospital room (home) was suitable and comfortable for living. |  |  |  |  |  |  |  |
| Necessary care was provided to help maintain the family’s health. |  |  |  |  |  |  |  |
| The amount of costs paid was reasonable. |  |  |  |  |  |  |  |
| My patient could be admitted to the hospital as soon as possible. |  |  |  |  |  |  |  |
